# The association between non-high-density lipoprotein cholesterol to high-density lipoprotein cholesterol ratio (NHHR) and suicidal ideation in adults: a population-based study in the United States

**DOI:** 10.1186/s12944-024-02012-4

**Published:** 2024-01-13

**Authors:** Guangwei Qing, Wenpeng Deng, Yuxin Zhou, Liyun Zheng, Yanlai Wang, Bo Wei

**Affiliations:** 1 Department of Psychiatry, Jiangxi Mental Hospital & Affiliated Mental Hospital of Nanchang University, Nanchang, Jiangxi 330029 China; 2 Third Clinical Medical College, Nanchang University, Nanchang, Jiangxi 330006 China; 3 Nanchang City Key Laboratory of Biological Psychiatry, Jiangxi Provincial Clinical Research Center on Mental Disorders, Jiangxi Mental Hospital, Nanchang, Jiangxi 330029 China

**Keywords:** NHHR, Lipid ratio, Suicidal ideation, NHANES, Cross-sectional study

## Abstract

**Background:**

The ratio of non-high-density lipoprotein cholesterol (non-HDL-C) to high-density lipoprotein cholesterol (HDL-C) (NHHR) serves as a reliable lipid indicator associated with atherogenic characteristics. Studies have indicated a potential connection between suicidality and lipid metabolism. This research aims to investigate any possible association between the NHHR and the emergence of suicidal ideation within the confines of the study.

**Methods:**

This study examined the association between NHHR levels and suicidal ideation using data from the National Health and Nutrition Examination Survey (NHANES), conducted in the United States spanning 2005 and 2016. Calculation of the NHHR corresponds to the proportion of HDL-C to Non-HDL-C. The Patient Health Questionnaire-9’s ninth question was implemented for assessing suicidal ideation. Using subgroup analysis, smooth curve fitting, and multivariate logistic regression analysis, the research was conducted.

**Results:**

Encompassing a cohort of 29,288 participants, the analysis identified that 3.82% of individuals reported suicidal ideation. After using multivariable logistic regression and thorough adjustments, elevated NHHR levels were significantly and positively associated with a heightened likelihood of suicidal ideation, according to the findings (odds ratio [OR] = 1.06; 95% confidence interval [CI]: 1.02–1.11; *P* = 0.0048). Despite extensive adjustment for various confounding factors, this relationship remained consistent. An inverted U-shaped curve was utilized to illustrate the link between NHHR and suicidal ideation among nonsmokers; the curve’s inflection point was situated at 7.80. Subgroup analysis and interaction tests (all *P* for interaction > 0.05) demonstrated that there was no significant influence of the following variables on this positive relationship: age, sex, race, body mass index, education level, married status, hypertension, diabetes, and smoking status.

**Conclusion:**

Significantly higher NHHR levels were associated with an elevated likelihood of suicidal ideation. Based on these results, it is probable that NHHR may serve as a predictive indicator of suicidal ideation, emphasizing its potential utility in risk assessment and preventive strategies.

## Background

Suicide, characterized as a fatality resulting from a purposeful act of self-directed harm, exhibits systematic variations influenced by factors such as age, gender, and the chosen method of self-harm [1]. Worldwide, suicidal behaviors significantly impact public health, with an estimated incidence of 11.4 suicides per 100,000 people and 804,000 suicide-related fatalities [2]. Suicidal ideation, playing a pivotal role as a significant predictor of both attempted and completed suicides [3], poses a considerable health burden due to its predictive relevance [4]. Therefore, allocating increased clinical attention to suicidal ideation is imperative.

The secondary messenger system of the brain heavily relies on cholesterol, closely linked to the actions of mood stabilizers and antidepressants [5]. This could potentially exert an indirect influence on the emergence of suicidal ideation. The intricate relationship that exists between lipid metabolism and suicidal ideation has been explored by multiple studies. The triglyceride-glucose (TyG) index and suicidal ideas were shown to be significantly associated in cross-sectional research involving 21,350 participants who were over the age of nineteen. However, with male individuals, no significant relationship was observed [6]. In a cross-sectional investigation, Hee-Young et al. observed a correlation between lower triglyceride levels and a decreased probability of experiencing suicidal ideation in a sample of 4557 Korean adults over the age of 65 [7]. Anhedonia was associated with lower LDL levels compared to equivalent control groups, but considerations of suicide were linked to more elevated HDL and cholesterol levels in a Chinese study including 287 untreated depressed patients [8].

Furthermore, research has shown that the ratio of non-high-density lipoprotein cholesterol (non-HDL-C) to HDL-C ratio (NHHR) serves as an independent risk indicator of depression in adults in the United States [9]. Approximately 90% of individuals with suicidal ideation have treatable psychological disorders, predominantly depression [10]. In the ongoing research into the association between psychological well-being and lipid metabolism, NHHR serves as a recently created composite indicator that assesses atherogenic lipid profiles and provides extensive insight into lipid particles that are both anti-atherogenic and atherogenic [11]. To determine the NHHR, non-HDL-C levels are divided by comparable HDL-C levels [12]. Previous research has shown that the NHHR exhibits superior diagnostic efficacy in comparison with standard lipid parameters in predicting the risk of cerebrovascular diseases, liver disease, insulin resistance, and metabolic syndrome [13–15]. Therefore, exploring the relationship between the NHHR and suicidal ideation may provide valuable insights into the intersection between lipid metabolism and mental health, prompting further investigation into preventive strategies and interventions.

Despite the growing body of evidence associating lipid metabolism to suicidal ideation, suicidal ideation, and NHHR have not been thoroughly studied in any previous studies. Thus, the primary goal for the research was to investigate if suicidal ideation and the NHHR were associated. It is hypothesized that an increased NHHR might be linked to an increased likelihood of suicidal ideation. By shedding light on the links between lipid metabolism and mental health, this research will contribute to resolving the knowledge deficit concerning the association between NHHR and suicide ideology. In essence, this study explores a newly discovered field concerning the potential predictive use of the NHHR for mental health outcomes and focuses on an innovative perspective of suicidal ideation and its association with lipid metabolism.

## Methods

### Study population

The National Health and Nutrition Examination Survey (NHANES), an investigation that collects demographic information on the health and nutrient intake of US citizens, is supervised and executed through the National Center for Health Statistics (NCHS). On account of this study’s design’s utilization of a stratified multistage probability sampling process, the samples included in NHANES exhibit ideal representativeness [16]. Participants undergo a health check in a mobile examination facility and a standardized in-home interview to assess their physical and medical conditions. Additional tests are conducted to gather pertinent laboratory data. The NCHS Research Ethics Review Board authorized ethical applications for NHANES involving human subjects, along with every participant has officially granted their informed consent. By visiting https://www.cdc.gov/nchs/nhanes/, the public can access all of the NHANES data.

**Fig. 1:**
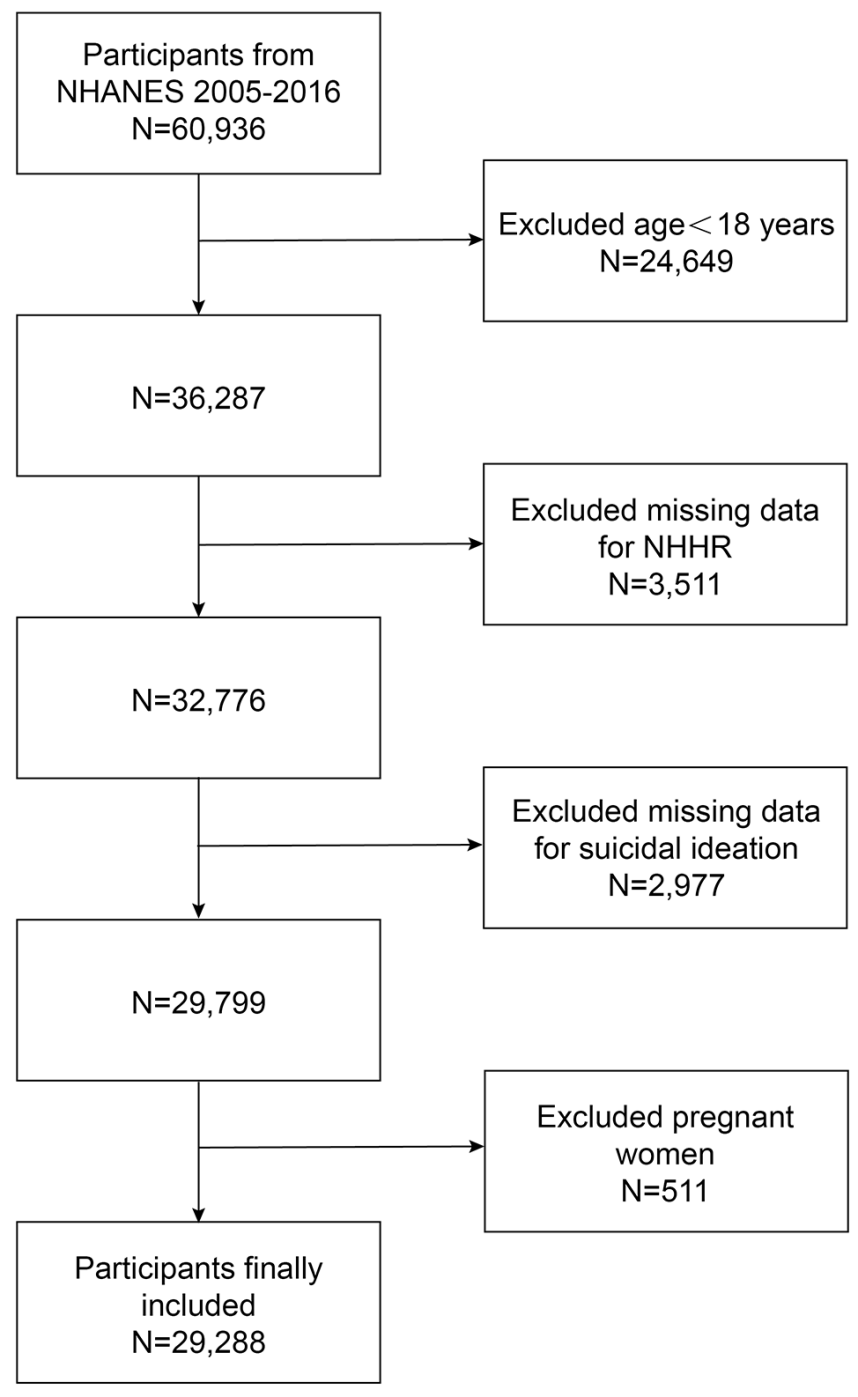
Flowchart of participant selection. NHANES, National Health and Nutrition Examination Survey; NHHR, non-high-density lipoprotein cholesterol to high-density lipoprotein cholesterol ratio

For this research, six cycles spanning 2005 to 2016 in NHANES were selected for the purpose of investigating the association between NHHR and suicidal ideation. This selection was based on the availability of comprehensive data on both the NHHR and suicidal ideation within four cycles. Initially, 60,936 participants were enrolled, with subsequent exclusions for individuals under 18 years of age (*n* = 24,649), those with missing NHHR data (*n* = 3,511), participants lacking data on suicidal ideation (*n* = 2,977), and pregnant individuals (*n* = 511). As a result, the final analytical cohort comprised 29,288 participants, as illustrated in Fig. 1.

### Assessment of NHHR

The NHHR serves as an independent variable in exposure assessment. The method outlined in prior studies was adopted to determine NHHR, specifically the Non-HDL-C/HDL-C ratio [17]. To derive non-HDL-C, obtained by subtracting HDL-C from total cholesterol (TC), lipid profiles of fasting individuals were analyzed. An automated biochemistry analyzer conducted an enzymatic test to evaluate TC and HDL-C levels. For TC concentration calculations, the research utilized both the Roche Cobas 6000 and Roche Modular P chemical analyzers in analytical procedures.

### Assessment of suicidal ideation

The application of the ninth item of the Patient Health Questionnaire-9 (PHQ-9) could be appropriate for the evaluation of suicidal ideation. The PHQ-9 comprises nine items and is utilized to ascertain whether an individual has exhibited depressive symptoms in the preceding two weeks [18]. Every single question in the survey has a score that spans from the assigned value “absent” at 0 to “nearly every day” at 3, which sums up to an overall total that falls between 0 and 27 [19]. A threshold of 10 is employed to identify the presence of depressive symptoms [20]. In the ninth item, respondents are asked, “Over the last two weeks, how frequently have you experienced thoughts of self-harm or the belief that your life would be better off ended?” “Not at all,” “several days,” “more than half the days,” and “nearly every day” are the available response options. For analytical purposes, all responses are categorized as either absent (no) or present at any frequency (yes) [21].

### Covariates

Potential variables that might introduce confounding effects in the relationship between NHHR and the occurrence of suicidal ideation were considered through the implementation of multivariate-adjusted models. Several variations in covariates were considered within this study’s analysis, encompassing gender (male or female), age (years), race, education level, waist circumference, body mass index (BMI), income-to-poverty ratio (PIR), marital status (married or living with a partner/widowed, divorced, separated, and never married), physical activity (inactive/active), depressive symptoms (non-depressive/depressive), TC (mg/dl), HDL-C (mg/dl), smoking status (smoker/non-smoker), diabetes, and hypertension. Each person’s BMI was classified as being under 25, between 25 and 30 kg/m2, and above 30 kg/m2, which are divided into the categories of normal weight, overweight, and obese, in that order. The operational definition of physical activity involves engaging in activities of moderate or vigorous intensity for a minimum duration of 10 continuous minutes outside occupational or transportation contexts. In contrast, physical inactivity was defined as involvement in the mentioned activities for less than 10 min [22]. The total quantity of dietary cholesterol ingested was determined from the averages of the two 24-hour dietary recall tests, utilizing comprehensive nutrient consumption data. For detailed information on the quantifiable processes of the study variables, www.cdc.gov/nchs/nhanes/ is the official website accessible to the public.

### Statistical analysis

The statistical analyses were performed following the protocols outlined by the Centers for Disease Control and Prevention (CDC). These guidelines prescribed the incorporation of relevant NHANES sample weights and the consideration of the complexities inherent in multistage cluster surveys. Standard deviations and means are shown for continuous data, while percentages are employed to depict categorical variables. A Student’s t-test with weights was employed to assess variations among groups based on the existence or lack of suicidal ideation. For evaluating associations between categories, a weighted chi-square test was utilized. By utilizing multivariate logistic regression, an independent association between NHHR and suicidal ideation in three distinct models was examined. Model 1 lacked covariate adjustments, meanwhile, Model 2 incorporated gender, age, and race adjustments. Gender, age, race, marital status, level of education, BMI, PIR, smoking status, diabetes, hypertension, physical activity, and dietary cholesterol were among the additional variables incorporated into Model 3. By implementing penalized spline smooth curve fitting and weighted generalized additive model (GAM) regression analysis, the non-linear association between NHHR and suicidal ideation was examined. Subgroup analyses were conducted using stratified multivariate regression analysis, with stratification based on sex, age, race, BMI, educational level, marital status, hypertension, diabetes, and smoking status. The log-likelihood ratio test model was employed for subgroup analysis, and statistical significance was determined at *P* < 0.05. The analytical procedures were implemented using R 3.4.3 (available at http://www.R-project.org) and Empower (available at www.empowerstats.com) as software applications.

## Results

29,288 individuals made up the study’s sample; 48.52% of them consisted of men and 51.48% were women. The research individuals’ average ages were 48.00 ± 18.70 years. Among them, 28,168 (96.18%) reported an absence of suicidal ideation, while 1,120 (3.82%) indicated exhibiting ideas of suicide. Significant distinctions were observed among the groups classified by the absence or presence of suicidal ideation regarding the following factors: education level, gender, race, marital status, dietary cholesterol, HDL-C, income-to-poverty ratio, smoking status, diabetes, hypertension, physical activity, depressive symptoms, and dietary cholesterol and waist circumference (*P* < 0.05). The attributes pertaining to those who were more likely to encounter suicidal ideas included being male, non-Hispanic White, widowed, divorced, separated, and never married, as well as having some college education or an AA degree and smoking. They also exhibited higher BMI levels, waist circumference, TC levels, and NHHR levels, and had higher rates of active physical activity and depression. However, they had lower rates of diabetes and hypertension, as well as lower household income, dietary cholesterol, and HDL-C levels within the research (*P* < 0.05). Individuals’ clinical and physiological characteristics are categorized according to whether or not they have suicidal ideation in Table 1.

**Table 1:** Characteristics of the study population

Characteristic	Total (*N* = 29,288)	Without suicidal ideation (*N* = 28,168)	With suicidal ideation (*N* = 1120)	*P*-value
Age(year)	48.00 ± 18.70	47.99 ± 18.72	48.12 ± 18.16	0.793
Gender (%)				< 0.001
Male	17,360 (48.52%)	14,421 (51.20%)	668 (59.64%)	
Female	18,416 (51.48%)	13,747 (48.80%)	452 (40.36%)	
Race(%)				< 0.001
Mexican American	4876 (16.65%)	4655 (16.53%)	221 (19.73%)	
Other Hispanic	2819 (9.63%)	2642 (9.38%)	177 (15.80%)	
Non-Hispanic White	12,770 (43.60%)	12,337 (43.80%)	433 (38.66%)	
Non-Hispanic Black	6124 (20.91%)	5915 (21.00%)	209 (18.66%)	
Other Race	2699 (9.22%)	2619 (9.30%)	80 (7.14%)	
Marital status(%)				< 0.001
Married or Living with Partner	16,537 (59.02%)	16,059 (59.59%)	478 (44.67%)	
Widowed, divorced, separated, and never married	11,483 (40.98%)	10,891 (40.41%)	592 (55.33%)	
Education level(%)				< 0.001
Less Than 9th Grade	2919 (10.59%)	2729 (10.30%)	190 (18.08%)	
9-11th Grade	4005 (14.53%)	3783 (14.27%)	222 (21.12%)	
High School Grad/GED or Equivalent	6310 (22.90%)	6070 (22.90%)	240 (22.84%)	
Some College or AA degree	8045 (29.20%)	7771 (29.32%)	274 (26.07%)	
College Graduate or above	6276 (22.78%)	6151 (23.21%)	125 (11.89%)	
Body mass index(kg/m^2^), (%)				< 0.001
< 25	8767 (30.23%)	8445 (30.27%)	322 (29.30%)	
25 to < 30	9536 (32.88%)	9220 (33.05%)	316 (28.75%)	
≥ 30	10,697 (36.89%)	10,236 (36.69%)	461 (41.95%)	
Waist circumference(cm)	98.60 ± 16.47	98.52 ± 16.40	100.70 ± 18.06	< 0.001
Income to poverty ratio	2.49 ± 1.63	2.52 ± 1.63	1.72 ± 1.38	< 0.001
Smoking status(%)				< 0.001
Yes	12,687 (45.21%)	12,097 (44.82%)	590 (55.09%)	
No	15,375 (54.79%)	14,894 (55.18%)	481 (44.91%)	
Diabetes(%)				< 0.001
Yes	3522 (12.03%)	3317 (11.78%)	205 (18.30%)	
No	25,124 (85.85%)	24,235 (86.10%)	889 (79.38%)	
Borderline	620 (2.12%)	594 (2.11%)	26 (2.32%)	
Hypertension(%)				< 0.001
Yes	10,095 (34.52%)	9618 (34.19%)	477 (42.74%)	
No	19,153 (65.48%)	18,514 (65.81%)	639 (57.26%)	
Physical activity(%)				< 0.001
Inactive	12,151 (48.66%)	11,832 (49.30%)	319 (32.92%)	
active	12,820 (51.34%)	12,170 (50.70%)	650 (67.08%)	
Depressive symptom(%)				< 0.001
Without depression	26,282 (89.74%)	25,947 (92.12%)	335 (29.91%)	
With depression	3006 (10.26%)	2221 (7.88%)	785 (70.09%)	
Dietary cholesterol (mg)	286.16 ± 187.71	286.74 ± 188.01	270.82 ± 178.99	0.003
TC (mg/dL)	191.91 ± 41.88	191.80 ± 41.73	194.88 ± 45.38	0.065
HDL-C (mg/dL)	52.74 ± 15.98	52.81 ± 15.98	51.05 ± 15.72	< 0.001
NHHR	2.93 ± 1.45	2.93 ± 1.45	3.14 ± 1.58	< 0.001

### The association between NHHR and suicidal ideation

The research results revealed that a positive association existed between elevated NHHR and an increased likelihood of experiencing suicidal ideation. This association existed in the initial unadjusted model and remained statistically significant in subsequent models, even after minimal and thorough adjustments were applied. With all adjustments implemented on Model 3 (OR = 1.06; 95% CI: 1.02–1.11; *P* = 0.0048), it was observed a 6% rise in the likelihood of suicidal ideation for each unit increase in NHHR. To further explore this relationship, it was categorized the continuous variable NHHR into discrete intervals (tertiles) for a sensitivity analysis. In the partially adjusted model (Model 2), tertile 3 exhibited a 20% higher probability of suicidal ideation compared to the lowest NHHR tertile (tertile 1) (OR = 1.20; 95% CI: 1.03–1.39; *P* = 0.0179). However, after extensive adjustments, the observed association (OR = 1.15; 95% CI: 0.94–1.41; *P* = 0.1751) did not reach statistical significance. Furthermore, none of the three models were able to significantly differentiate between tertile 1 and tertile 2 (Table 2).

**Table 2:** The association between NHHR and suicidal ideation

	Crude Model (Model 1)	Partially Adjusted Model (Model 2)	Fully Adjusted Model (Model 3)
	OR (95% CI) *p*-value	OR (95% CI) *p*-value	OR (95% CI) *p*-value
NHHR	1.09 (1.06, 1.13) < 0.0001	1.08 (1.04, 1.12) < 0.0001	1.06 (1.02, 1.11) 0.0048
NHHR Tertiles			
Tertile 1	Reference	Reference	Reference
Tertile 2	1.04 (0.90, 1.22) 0.5819	0.98 (0.84, 1.14) 0.7553	0.93 (0.76, 1.15) 0.5030
Tertile 3	1.30 (1.13, 1.51) 0.0004	1.20 (1.03, 1.39) 0.0179	1.15 (0.94, 1.41) 0.1751
P for trend	1.12 (1.05, 1.19) 0.0002	1.08 (1.02, 1.15) 0.0083	1.07 (0.99, 1.17) 0.0857

Model 1, no covariates were adjusted. Model 2, age, gender, and race were adjusted. Model 3, age, gender, race, marital status, education level, BMI, income-to-poverty ratio, smoking status, diabetes, hypertension, physical activity, and dietary cholesterol were adjusted. 95% CI, 95% confidence interval; OR, odds ratio

### A nonlinear relationship between NHHR and suicidal ideation

**Fig. 2:**
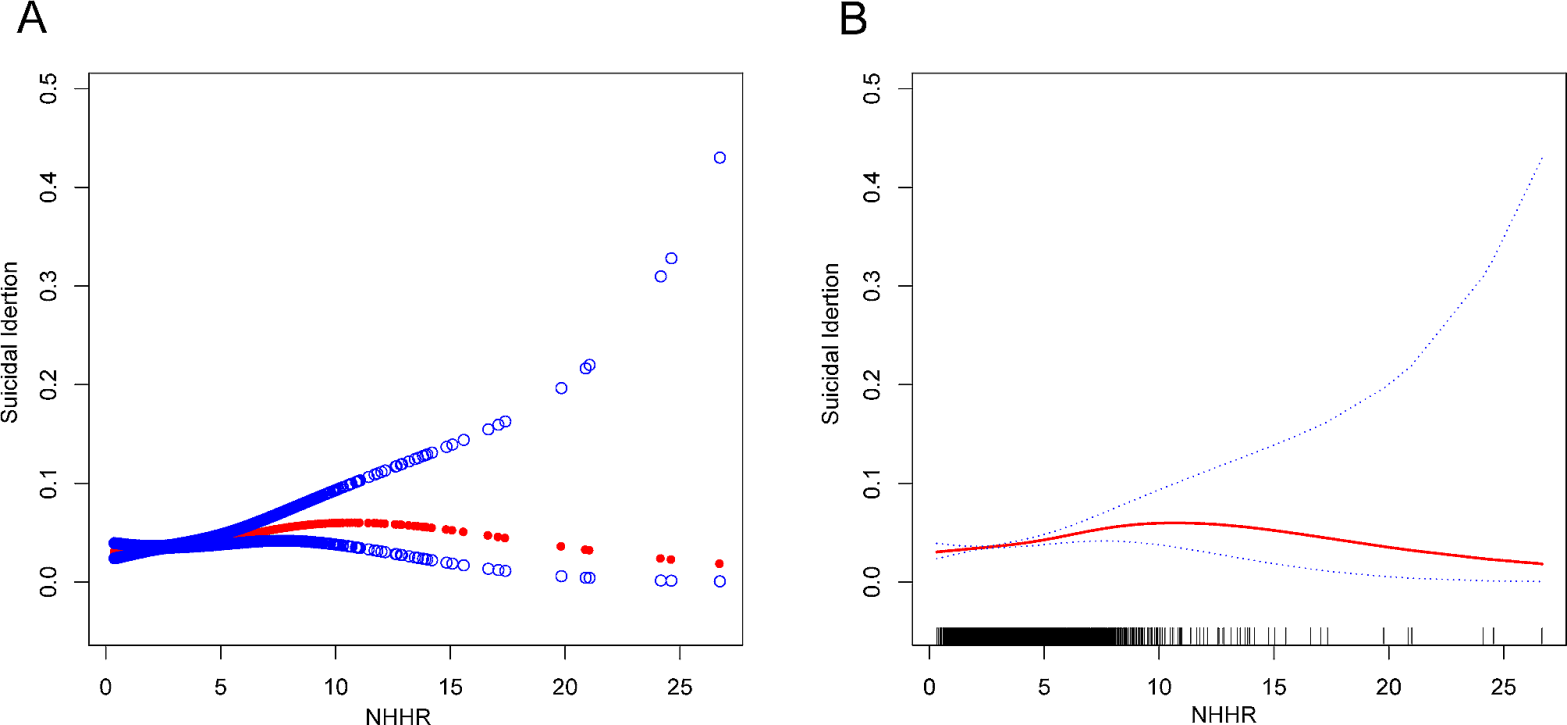
The association between NHHR and suicidal ideation. (**A**) The solid red line represents the smooth curve fit between variables. (**B**) Blue bands represent the 95% confidence interval from the fit. NHHR, non-high-density lipoprotein cholesterol to high-density lipoprotein cholesterol ratio

**Fig. 3:**
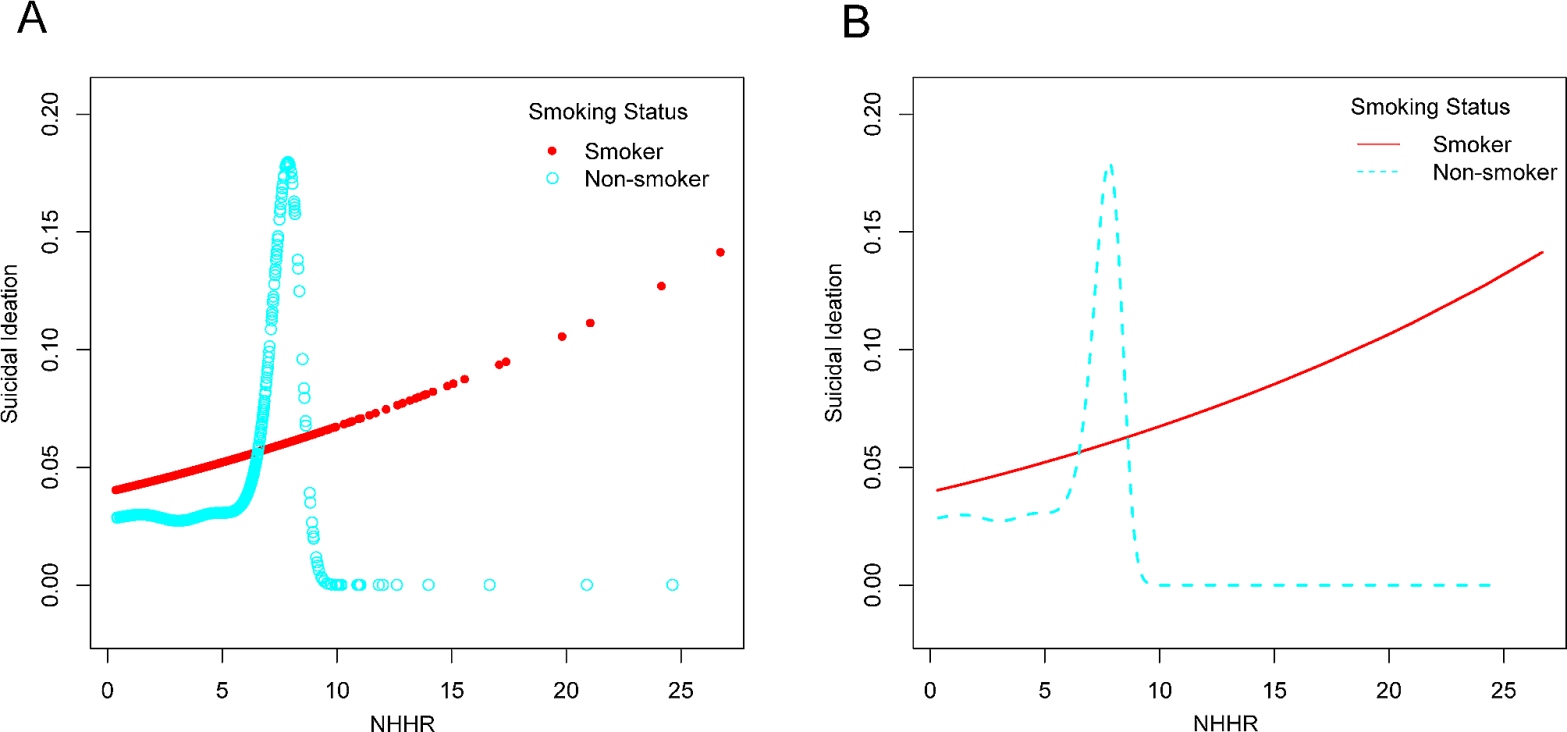
The association between NHHR and suicidal ideation is stratified by smoking status. NHHR, non-high-density lipoprotein cholesterol to high-density lipoprotein cholesterol ratio

Through the application of smooth curve fits and weighted generalized additive models, an in-depth analysis of the nonlinear relationship between NHHR levels and suicidal ideation was undertaken for this study. A non-linear relationship was revealed by the results, as illustrated in Fig. 2. Upon further examination, after stratifying by smoking status, it was observed an inverted U-shaped curve with an inflection point at 7.80 within the non-smoker subgroup (Fig. 3; Table 3). This pattern persisted even when accounting for the same covariates.

**Table 3:** The threshold effect of NHHR on suicidal ideation stratified by smoking status was analyzed using a two-part linear regression model

	Smoking status	
	Yes	No
Fitting by standard linear model		
OR (95% CI)	1.10 (0.96, 1.26)	1.08 (1.00, 1.16)
*P*-value	0.1596	0.0502
Fitting by two-piecewise linear model		
Breakpoint (K)	2.42	7.80
OR1(<K)	2.03 (0.83, 4.97) 0.1211	1.14 (1.04, 1.25) 0.0054
OR2(>K)	1.04 (0.87, 1.24) 0.6749	0.47 (0.11, 2.09) 0.3236
Logarithmic likelihood ratio test *P*-value	0.150	0.028

Age, gender, race, marital status, education level, BMI, income-to-poverty ratio, smoking status, diabetes, hypertension, physical activity, and dietary cholesterol were adjusted. 95% CI, 95% confidence interval; OR, odds ratio

### Subgroup analysis

The robustness of the association between NHHR and suicidal ideation was evaluated using subgroup analysis (Table 4). Despite this, the *p*-values for the interaction (all *P* > 0.05) indicated no statistically significant association, implying that variables encompassing age, gender, race, BMI, education level, marital status, hypertension, diabetes, and smoking status did not influence the association. Notably, the findings continuously indicate a significant link between NHHR and suicidal ideation even controlling for major demographic variables comprising age, sex, race, BMI, education level, marital status, hypertension, diabetes, and smoking status. This suggests the potential relevance of this association across diverse population settings.

**Table 4:** Association between NHHR and suicidal ideation in subgroups

Subgroup		OR(95%CI)	*P* for interaction
Age(year)			0.4618
< 50	*N* = 9156	1.05 (0.98, 1.12)	
≥ 50	*N* = 9346	1.08 (1.02, 1.15)	
Gender			0.3642
Male	*N* = 9526	1.05 (0.99, 1.11)	
Female	*N* = 8992	1.09 (1.02, 1.17)	
Race			0.5036
Mexican American	*N* = 2669	1.08 (0.97, 1.20)	
Other Hispanic	*N* = 1841	1.16 (1.02, 1.32)	
Non-Hispanic White	*N* = 8613	1.06 (1.00, 1.13)	
Non-Hispanic Black	*N* = 3676	1.06 (0.93, 1.22)	
Other Race	*N* = 1719	0.94 (0.76, 1.16)	
BMI(kg/m2)			0.5389
< 25	*N* = 5155	1.09 (0.96, 1.24)	
25–30	*N* = 6199	1.10 (1.01, 1.20)	
> 30	*N* = 7315	1.04 (0.98, 1.11)	
Education level			
Less Than 9th Grade	*N* = 1610	1.07 (0.95, 1.22)	
9-11th Grade	*N* = 2535	1.03 (0.93, 1.13)	
High School Grad/GED or Equivalent	*N* = 4228	1.08 (0.99, 1.18)	
Some College or AA degree	*N* = 5537	1.02 (0.92, 1.12)	
College Graduate or above	*N* = 4600	1.21 (1.06, 1.38)	
Marital status			0.5930
Married/living with partner	*N* = 11,162	1.05 (0.99, 1.12)	
Widowed/divorced/separated/ Never married	*N* = 7343	1.08 (1.01, 1.15)	
Hypertension			0.9299
Yes	*N* = 6831	1.06 (1.00, 1.13)	
No	*N* = 11,670	1.06 (0.99, 1.13)	
Diabetes			0.1128
Yes	*N* = 2392	1.03 (0.93, 1.13)	
No	*N* = 15,688	1.09 (1.03, 1.15)	
Borderline	*N* = 430	0.80 (0.55, 1.15)	
Smoking status			0.7194
Yes	*N* = 8344	1.06 (1.00, 1.12)	
No	*N* = 10,158	1.08 (1.00, 1.16)	

The results show that the subgroup analysis was adjusted for all presented covariates except the effect modifier. 95% CI, 95% confidence interval; OR, odds ratio

## Discussion

In this comprehensive study involving 29,288 adults, the findings indicate that individuals with elevated NHHR scores are more likely to experience suicidal ideation. This association holds true across various subgroups, including age, sex, race, BMI, educational level, marital status, hypertension, diabetes, and smoking status. These results are consistent across diverse population settings, as demonstrated by subgroup analysis and interaction tests. Notably, within the non-smoker group, an inverted U-shaped association was discovered between NHHR and suicidal ideation, characterized by an inflection point at 7.80. In accordance with the results, which can be speculated that NHHR may serve as a predictor of suicidal ideation and that regulating lipid levels as determined by NHHR could reduce suicidal ideation and associated behaviors.

From the utmost of present understanding, this research signifies the relationship’s primary investigation between NHHR and suicidal ideation. Growing evidence supports the notion that NHHR is a superior indicator of lipid-related disorder risk [23–25]. While empirical research examining the relationship between NHHR levels and ideation of suicide is lacking, a wealth of literature exists exploring the links between suicidal ideation and various lipid-related factors. In a cross-sectional investigation involving 13,772 adults in Korea, Hana et al. identified a significant association, indicating that reduced levels of LDL-C were linked to an elevated likelihood of suicidal thoughts for male individuals above the age of 19 [5]. A potential association was identified by Bałażej et al. between increased levels of TC and LDL and the occurrence of suicidal thoughts in females who were undergoing their initial episode of schizophrenia [26]. A retrospective cohort investigation involving 73 outpatients diagnosed with major depressive disorder suggested a noteworthy reduction in TG levels within the cohort exhibiting suicidal ideation, contrasting with individuals lacking such notions [27]. Suicidal ideation and completed suicides are associated with decreased blood lipid levels, particularly TC, as stated by Shunquan et al. in a meta-analysis involving 65 epidemiological studies [28]. While these findings do not directly present evidence, they indirectly support that NHHR levels are positively associated with suicidal ideation, contributing to the growing body of literature examining the association between profiles of lipids and suicidal tendencies through the use of novel lipid characteristics.

The NHHR, a recently amalgamated metric reflecting atherogenic lipid composition [29], surpasses conventional lipid parameters in evaluating atherosclerosis extent [30]. Kwok RM states that compared to other lipid indicators, the NHHR has a more robust predictive ability for non-alcoholic fatty liver disease (NAFLD) [31]. According to Lin D’s study, the NHHR is a reliable diagnostic instrument for assessing insulin resistance. Compared to normal lipid inspections, this metric demonstrated superior accuracy in predicting conditions associated with the development of diabetes [32]. In summary, the NHHR has demonstrated exceptional predictive efficacy in a variety of studies. Furthermore, the NHHR is a widely accessible method distinguished by its noninvasive nature, ease of accessibility, and cost-effectiveness, presenting promising prospects for clinical implementation.

Various perspectives offer explanations for the association between lipid metabolism and thoughts of suicide. Some theories propose that reduced cholesterol levels may influence the microviscosity of serotonin receptors, impacting serotonin activity and contributing to impulsive and suicidal behaviors [33]. An elevation in the ratio of n-6/n-3 PUFAs which is linked to the induction of a pro-inflammatory state, according to mechanisms regarding the balance of polyunsaturated fatty acids (PUFAs) and inflammation [34]. Further evidence substantiates this association, as studies have linked suicidal ideation with pro-inflammatory cytokines, including IL-6 [35]. Collectively, these findings suggest a potential key component in the pathophysiological model of suicide behavior. According to a theory proposed by Penttinen et al., increased cytokine production, specifically interleukin-2 (IL-2), leads to higher total blood cholesterol and lower serum HDL cholesterol, affecting melatonin release and increasing impulsivity and suicide risk [36, 37]. Considering variations in dietary patterns within the target demographic, one plausible interpretation is that PUFA consumption correlates with reduced TG levels, potentially mitigating the risk of suicidal ideation [38, 39]. Therefore, employing the NHHR as a means to assess the non-HDL-C proportion in patients could serve as a more effective tool for evaluating the impact that lipid metabolism has on the occurrence of suicidal ideation.

### Strengths and limitations

This study exhibits several strengths. Firstly, NHANES data was utilized, representing a comprehensive and nationally representative sample obtained using a consistent procedure with a sufficient sample size [40]. Additionally, the research meticulously controlled for confounding covariates, selecting them primarily based on prior investigations that evaluated the association between suicidal ideation and various exposure variables. This approach was undertaken to enhance the reliability and validity of the results. However, it’s essential to acknowledge the inherent limits of this research. First, the assessment of suicidal ideation relied on personal interviews, introducing an inevitable recall bias. Second, though the PHQ-9’s ninth item has been used in prior research to measure suicide ideation, its extensive definition—which includes non-suicidal self-harm—may affect how the study evaluates the item’s association with suicidal ideation. Third, comprehensive validation of the PHQ-9’s utility in assessing suicidal ideation among the general public is lacking. Nonetheless, when it comes to basic internal medicine primary care, PHQ-9 possesses strengthened specificity and sensitivity. Fourth, the cholesterol data analyzed in this study were derived from fasting individuals, with non-fasting data remaining unexplored. Discrepancies in the laboratory testing protocols may introduce potential biases. Fifth, reverse causality was a possibility since the study employed a design with a cross-sectional approach, which hinders the ability to establish a causal relationship. Hence, there remains a necessity for prospective investigations encompassing larger sample sizes to elucidate the causative relationship. Meanwhile, despite adjusting for certain potential covariates, fully mitigating potential confounding factors beyond those adjusted remains elusive within the scope of the research.

## Conclusion

Based on the analysis conducted, a notable association was identified between suicidal ideation and higher NHHR scores, emphasizing the potential clinical relevance of lipid metabolism in mental health. Recognizing NHHR as a predictive indicator suggests a proactive two-step approach in routine lipid profile screenings: identifying potential mental health risks through abnormal NHHR levels and conducting comprehensive mental health assessments. This finding provides a valuable tool for early suicide risk detection, particularly in psychiatric care, allowing healthcare professionals to closely monitor mental health, support personalized interventions, and enhance overall psychiatric care effectiveness.

## Data Availability

In this study, publicly accessible datasets were examined. These data can be found here: ((https://wwwn.cdc.gov/nchs/nhanes/analyticguidelines.aspx, accessed on 1 November 2022).

## Abbreviations

non-HDL-C: Non-high-density lipoprotein cholesterol
HDL-C: High-density lipoprotein cholesterol
NHHR: Non-HDL-C and HDL-C ratio
PIR: Poverty-to-income ratio
BMI: Body mass index
TC: Cholesterol
